# The clinical course over the first year of Whiplash Associated Disorders (WAD): pain-related disability predicts outcome in a mildly affected sample

**DOI:** 10.1186/1471-2474-14-361

**Published:** 2013-12-21

**Authors:** Pernilla Åsenlöf, Annika Bring, Anne Söderlund

**Affiliations:** 1 Department of Neuroscience, Uppsala University, BMC, Box 593, S-751 24 Uppsala, Sweden; 2 School of Health, Care and Social Welfare, Physiotherapy, Mälardalen University, P.O.Box 883, SE-721 23 Västerås, Sweden

**Keywords:** Acute whiplash associated disorders, Whiplash injury, Prognosis, Pain-related disability, Risk factors

## Abstract

**Background:**

Different recovery patterns are reported for those befallen a whip-lash injury, but little is known about the variability within subgroups. The aims were (1) to compare a self-selected mildly affected sample (MILD) with a self-selected moderately to severely affected sample (MOD/SEV) with regard to background characteristics and pain-related disability, pain intensity, functional self-efficacy, fear of movement/(re)injury, pain catastrophising, post-traumatic stress symptoms in the acute stage (at baseline), (2) to study the development over the first year after the accident for the above listed clinical variables in the MILD sample, and (3) to study the validity of a prediction model including baseline levels of clinical variables on pain-related disability one year after baseline assessments.

**Methods:**

The study had a prospective and correlative design. Ninety-eight participants were consecutively selected. Inclusion criteria; age 18 to 65 years, WAD grade I-II, Swedish language skills, and subjective report of not being in need of treatment due to mild symptoms. A multivariate linear regression model was applied for the prediction analysis.

**Results:**

The MILD sample was less affected in all study variables compared to the MOD/SEV sample. Pain-related disability, pain catastrophising, and post-traumatic stress symptoms decreased over the first year after the accident, whereas functional self-efficacy and fear of movement/(re)injury increased. Pain intensity was stable. Pain-related disability at baseline emerged as the only statistically significant predictor of pain-related disability one year after the accident (Adj r^2^ = 0.67).

**Conclusion:**

A good prognosis over the first year is expected for the majority of individuals with WAD grade I or II who decline treatment due to mild symptoms. The prediction model was not valid in the MILD sample except for the contribution of pain-related disability. An implication is that early observations of individuals with elevated levels of pain-related disability are warranted, although they may decline treatment.

## Background

About 50% fail to recover after a whiplash occurrence [1] why prospective studies on prognostic factors for recovery are urgent. An early identification of individual’s prognosis according to evidence-based clinical prediction rules [2] may contribute to tailored management in accordance with identified factors in the acute and subacute phases. However, the current evidence is not sufficiently robust since most studies are exploratory in nature, and few of identified prognostic models have been externally validated in new and independent cohorts [3].

Based on up-to-date evidence initial high pain intensity [4,5] and pain-related disability [1,4] are the most consistent prognostic factors for prolonged disability. There is preliminary evidence that central hyper-excitability or signs of central sensitization predict recovery [3]. Among psychological factors there is preliminary evidence that low self-efficacy, symptoms of post-traumatic stress, pain catastrophising, depressed mood, and fear of movement/(re)injury have predictive value [1,6]. Recent studies propose that functional self-efficacy [7] and fear of movement/(re)injury [8] mediates the relationship between pain and disability.

A limitation with current evidence is the lack of reports on how prognostic factors identified in the acute phase develop over time [3], and the significance of changes, or lack of changes in these factors, for recovery. Cohort studies indicate that most recovery occurs in the first 2 to 3 months after the accident [4], and different recovery patterns have been reported for subgroups with (1) initial mild and negligible pain, (2) initial moderate pain and pain-related disability, and (3) initial severe pain and pain-related disability [9]. Nevertheless, a common clinical apprehension built on experiences from physicians and physiotherapists is that individuals who report mild pain in the acute phase do to some extent return later to health care with complaints of increased symptoms and pain-related disability. Since there may be variation not yet uncovered within each of the subgroups a new approach would be to study the development over the first year after the injury and validate established prognostic factors for each subgroup separately. This could add essential knowledge on prognostic factors for recovery as well as prolonged disability.

During recruitment to a recent randomised controlled trial (RCT) [10] aiming to evaluate effects of a psychologically informed early management intervention after whiplash, we identified individuals declining participation due to mild symptoms of pain and disability 2 to 4 weeks after the accident. These individuals were asked for participation in the current longitudinal study which aimed to compare a self-selected mildly affected sample (MILD) with a self-selected moderately to severely affected sample (MOD/SEV) with regard to background characteristics and pain-related disability, pain intensity, functional self-efficacy, fear of movement/(re)injury, pain catastrophising, post-traumatic stress symptoms in the acute stage (at baseline). For the MILD sample we also aimed to study the development over the first year after the accident for the above listed clinical variables including the proportion of participants who reported a clinically relevant deterioration in pain-related disability. Finally, the validity of a prediction model including baseline levels of pain-related disability, pain intensity, functional self-efficacy, fear of movement/(re)injury, pain catastrophising, post-traumatic stress symptoms on pain-related disability 12 months after baseline assessment was studied.

## Methods

### Study design

The study had a prospective, longitudinal, and correlative design to study changes over time and prediction. Assessments were performed at baseline (2–4 weeks after the accident) and at 3, 6, and 12 months follow-ups. In addition, a cross-sectional comparison with another subsample was done at baseline. According to Swedish law studies using questionnaires without any intervention were not required to be reviewed by the ethic board at this point in time. However, The Regional Ethics Committee in Uppsala, Sweden approved the study protocol (2005:098). Information to participants, obtainment of consent, and other research procedures adhered to the Declaration of Helsinki.

### Setting and participants

**Figure 1:**
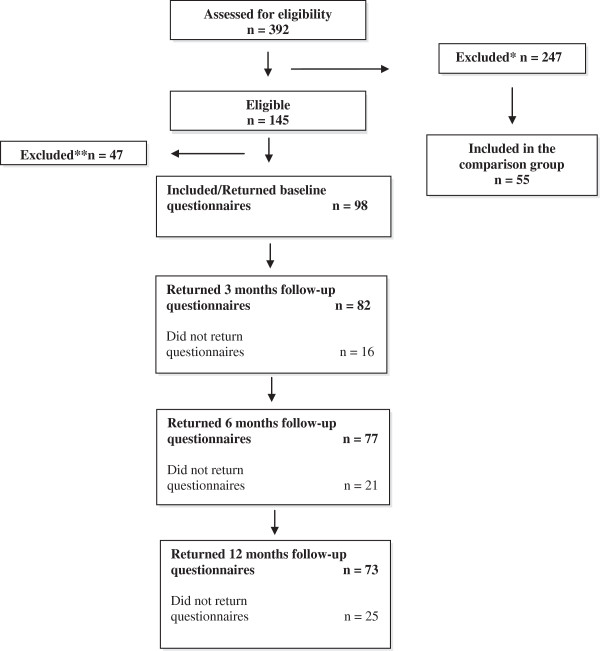
Diagram over the participant flow through recruitment, baseline assessment, 3-, 6- and 12-month follow-ups.

Ninety-eight participants were recruited from the emergency wards at two hospitals in Uppsala (University hospital) and Västerås (Regional county hospital) in Sweden between January 2007 and December 2009. Follow-ups were completed in December 2010. Eligibility criteria were; age 18 to 65 years, fulfilled criteria for the diagnosis of WAD grade I and II [11] established by a physician on the emergency ward within 72 hours from the accident, satisfactory Swedish language skills, and subjective report of not being in need of further treatment due to mild pain and disability 2–4 weeks after the accident. For more details is referred to Figure 1.

The sample size was determined by the number of patients giving informed consent to be contacted for research purposes within two weeks from the accident and subjectively reported being in no need of treatment due to mild pain and disability. We estimated a priori that a sample size of 98 participants would allow for testing regression with 6 predictors when assuming a medium-size relationship between independent and dependent variables and α = 0.05 and β = 0.20 [12].

### Selection and procedures

A consecutive selection was done meaning that all individuals attending the emergency wards during the scheduled time period underwent a physical examination to establish the WAD grade. Those eligible i.e. WAD grade I and II, received oral and written information about the RCT from an emergency nurse. They were given standardized, written self-management instructions for handling of common physical symptoms in WAD [13]. Within two weeks from the accident, individuals giving consent for being contacted by the study co-ordinator (second author) were contacted by telephone. Those who declined participation in the RCT due to no need of further treatment and mild residual symptoms were informed about the current study. Provided verbal agreement, written information and baseline measures were distributed by ordinary mail. Participants were encouraged to return the questionnaires immediately, but no later than 4 weeks after the accident for the reason of capturing baseline data from the acute stage. Forty of 145 eligible individuals failed to return baseline questionnaires despite two reminders. The mail procedure was repeated at 3, 6, and 12 months for all participants who returned the baseline questionnaires (Figure 1).

### Variables and measures

*Pain-related disability* was measured with the Swedish version of The Pain Disability Index (PDI) [14-17] that is a 7-item inventory designed to measure interference with role-functioning due to persistent pain. A general disability score ranging from 0 to 70 was calculated by summing scores of the seven items. Higher scores indicate higher disability. Acceptable psychometric properties have been reported for samples with persistent pain and WAD [14,15,18].

*Pain intensity* was operationalised as the average pain intensity experienced over the past two weeks, which was scored on a numerical rating scale (NRS) with anchors 0 (no pain) and 10 (worst pain imaginable/unbearable pain). The validity of NRS for pain intensity ratings is well documented and findings include positive, significant correlations with other measures of pain intensity [19].

*Self-efficacy in performing common everyday life activities* (functional self-efficacy) was measured by the Swedish version of The Self-Efficacy Scale (SES) [17,20]. The SES measures the strength of perceived self-efficacy in performing 20 common everyday life activities. A general self-efficacy score ranging from 0 to 200 was calculated computed by summing ratings of the 20 activities. Higher scores indicate higher self-efficacy. The Swedish version of SES has shown good reliability in patients with whiplash associated disorder WAD [18].

*Fear of movement and (re)injury* was measured by the Swedish version of the Tampa Scale of Kinesiophobia (TSK) [17,21]. A total score ranging from 17 to 68 was calculated where a higher total sum indicates more fear. The Swedish version of TSK has shown good reliability in patients with whiplash associated disorders [22].

*Pain catastrophising* was measured with the catastrophising subscale (6 items) from the Coping Strategies Questionnaire (CSQ) [23]. The sum of the 6 items was calculated to a sum score ranging from 0 to 36. Higher scores indicate higher frequency of catastrophic thinking. The Swedish version of CSQ has shown high internal consistency [24].

*Post-traumatic stress symptoms* was measured with the Impact of Event Scale (IES) [25]. The IES consists of 15 in which the patient is asked to report the occurrence of symptoms during the past seven days on four-point scales. High values indicate severe symptoms. A total IES-score was calculated, ranging from 0 through 75. The IES has been reported a valid measure of post traumatic stress reactions [26].

### Data management and statistical analysis

All data were analysed in the IBM SPSS Statistics© version 20.0. Included in the analyses were those with completed questionnaires from all time points. To avoid ‘mass imputation’, it was decided to exclude questionnaires where >25% of the items were missing. Missing values within the separate questionnaires were substituted with the median of each individual’s observed item scores. The total amount of questionnaires with occasional missing items for all measures during all assessments were n = 7. Absolute p-values are reported and the level for statistical significance was set at ≤ .05. The two samples were described and compared using descriptive statistics, chi-square tests and Mann Whitney U tests. The Friedman test was used to analyse statistical changes over time. The cut-off for a clinically relevant deterioration in pain-related disability over the first year was set to ≥ 11 points on the PDI according to a previous study [27]. Pearson’s product–moment correlation (r) was used to examine the associations between the outcome (pain related disability) assessed at 12 months follow-up, and the potential predictors assessed at baseline. Core assumptions of linearity were checked before performing the linear regression analysis i.e. independence of the residuals, normally distributed residuals and constant variance of the residuals. Variables were then statistically checked for multicollinearity, which resulted in variance inflated factors (VIF) between 1.4 and 1.8. Hence no severe multicollinearity was supposed to hazard the planned regression models. Multiple linear regression analysis with backward selection was performed to regress pain-related disability at the 12-month follow-up (y1:2) on baseline assessments of pain-related disability (y1:1), pain intensity (y2:1), functional self-efficacy (y3:1), fear of movement/(re)injury (y4:1), pain catastrophising (y5:1), and post-traumatic stress symptoms (y6:1). A forward selection was also performed. Both methods resulted in equal results, and the backward selection method is presented in the results section. Finally, a diagnostic check of the distribution of the residuals was done with the Cook’s distance tests. One participant had an extreme Cook’s distance (4.7) and was excluded from further analyses.

## Results

Three-hundred-and-ninety-two individuals with acute WAD were assessed for eligibility, whereof 145 fulfilled inclusion criteria for this study. Finally included were 98 participants, of who 73 provided data at the 12-month follow-up. Those who did not provide any 12-month data were not included in the regression analysis. They did not differ statistically from those included with respect to any of the study variables. See Figure 1.

Returned questionnaires with >25% missing items in an individual questionnaire were excluded according to the a priori criterion. The number of excluded questionnaires was at baseline (n = 2), at 3-month follow-up (n = 3), at 6-month follow-up (n = 1), and at 12-month follow-up (n = 1).

The background characteristics of the (MILD) sample are presented in Table 1 together with data for the comparison sample (MOD/SEV).

**Table 1:** Participant characteristics and baseline values of variables included in the regression analysis

	**Mildly affected sample MILD**	**Moderate/severely affected sample MOD/SEV**	**Test of statistical difference between groups**
			p-value
**Gender**	(n = 98)	(n = 55)	.087 (Chi^2^ = 2.924, df = 1)
Female	52 (53.1%)	37 (67.3%)	
Male	46 (46.9%)	18 (32.7%)	
**Age (years)**	(n = 98)	(n = 55)	.473 (t = -.72, df = 121)
Mean (*standard deviation*)	34.4 (11.4)	35.7 (10.3)	
**WAD-grade**	(n = 98)	(n = 55)	.004 (Chi^2^ = 8.089, df = 1)
I	48 (49.0%)	14 (25.5%)	
II	50 (51.0%)	41 (74.5%)	
**Marital status**	(n = 95)	(n = 54)	.158 (Chi^2^ = 3.685, df = 2)
Married or cohabitants	50 (52.6%)	37 (67.3%)	
Single	36 (37.9%)	16 (29.1%)	
Living with parents	9 (9.5%)	2 (3.6%)	
**Education**	(n = 94)	(n = 54)	.56 (Chi^2^ = 7.579, df = 3)
Elementary school	13 (13.3%)	3 (5.5%)	
High school	46 (46.9%)	25 (45.5%)	
University	35 (37.2%)	27 (50.0%)	
**Physical activity level before accident**	(n = 98)	(n = 55)	<.001 (Chi^2^ = 21.2, df = 3)
≥5 times/week	18 (18.4%)	22 (40.0%)	
2–4 times/week	39 (39.8%)	23 (41.8%)	
0–1 times/week	41 (41.8%)	7 (12–7)%)	
Never	0	3 (5.5%)	
**Health status before accident**	(n = 98)	(n = 55)	.019 (Chi^2^ = 9.907, df = 3)
Very good	41 (41.8%)	23 (41.8%)	
Good	53 (54.1%)	22 (40.0%)	
Somewhat good	4 (4.1%)	8 (14.5%)	
Bad	0	2 (3.6%)	
**Depressed mood before accident**	(n = 94)	(n = 55)	.164 (Chi^2^ = 3.613, df = 2)
Never	77 (78.6%)	14 (73.7%)	
Sometimes	17 (17.3%)	4 (21.1%)	
Often	0	1 (1.8%)	
**Previous road accident, but no remaining symptoms**	(n = 95)	(n = 55)	.
Yes	9 (9.2%)	12 (21.8%)	036
No	86 (87.8%)	43 (78.2%)	(Chi^2^ = 4.409, df = 1)
**PDI**	(n = 98)	(n = 55)	<.001
Median (IQR)	3 (4)	21 (12)	(z = -8.2)
**Pain intensity** NRS 0–10	(n = 94)	(n = 55)	<.001
Median (IQR)	2 (7)	5 (4)	(z = -9)
**SES**	(n = 97)	(n = 55)	<.001
Median (IQR)	187 (20)	162 (35)	(z = -7.4)
**TSK**	(n = 97)	(n = 55)	<.001
Median (IQR)	26.5 (6)	34 (30)	(z = -5.8)
**CAT**	(n = 98)	(n = 55)	.009
Median (IQR)	2 (20)	7 (19)	(z = -2.6)
**IES**	(n = 98)	(n = 55)	<.001
Median (IQR)	23 (14)	35 (28)	(z = -6.4)

PDI = Pain Disability Index (0-70), low scores indicate low disability.

Pain intensity (3 items 0-10), low scores indicate low pain intensity, control (1 item 0-10), low scores indicate low control.

SES = Self-Efficacy Scale (0-200), low scores indicate low efficacy expectations.

TSK = The Tampa Scale of Kinesiphobia (17-68), low scores indicate low fear.

CAT = The Coping Strategies Questionnaire (0-36). High scores indicate higher frequency of catastrophic thinking.

IES = Impact of Event Scale (0-75). High scores indicate severe symptoms.

The MILD sample had a significantly less proportion of individuals with WAD-grade II compared to the MOD/SEV sample. They reported a significantly lower level of physical activity and better health status before the accident. A higher proportion of the MOD/SEV sample had experienced a previous road accident.

The MILD sample reported lower levels of pain-related disability, pain intensity, fear of movement/(re)injury, pain catastrophising, post traumatic stress symptoms, and higher level of functional self-efficacy compared to the MOD/SEV sample (Table 1).

From baseline to the 12-month follow-up pain-related disability, pain catastrophising, and post traumatic stress symptoms statistically decreased in the MILD sample, whereas functional self-efficacy and fear of movement/(re)injury increased. Pain intensity was stable. See Table 2.

**Table 2:** Analyses of changes over the first year after whip-lash occurrence (Friedman test)

**Outcome**	**Baseline**	**3-month follow-up**	**6-month follow-up**	**12-month follow-up**	**p-value**
	**Median (IQR)**	**Median (IQR)**	**Median (IQR)**	**Median (IQR)**	**(Chi**^**2**^**, df)**
**PDI n = 57**	3 (5)	2 (5)	2 (5)	1 (6)	<.001 (24.7, 3)
**Pain Intensity n = 58**	2 (2)	1 (2)	1(2)	1 (2)	.31 (3.5, 3)
**SES n = 57**	188 (21)	190 (27)	192 (23)	191 (27)	<.001 (26.4, 3)
**TSK n = 55**	27 (7)	28 (6)	28 (6)	29 (7)	.007 (12.0, 3)
**CAT n = 57**	4 (10)	3 (9)	4 (10)	2 (7)	<.001 (20.8, 3)
**IES n = 56**	24 (17)	21.5 (16)	21 (15)	22 (15)	<.001 (33.5, 3)

*Note:* N is based on those participants with completed questionnaires from all time points.

PDI = Pain Disability Index (0–70), low scores indicate low disability.

Pain intensity (3 items 0–10), low scores indicate low pain intensity, control (1 item 0–10), low scores indicate low control.

SES = Self-Efficacy Scale (0–200), low scores indicate low efficacy expectations.

TSK = The Tampa Scale of Kinesiphobia (17–68), low scores indicate low fear.

CAT = The Coping Strategies Questionnaire (0–36). High scores indicate higher frequency of catastrophic thinking.

IES = Impact of Event Scale (0–75). High scores indicate severe symptoms.

Four participants deteriorated 11 or more points on the PDI from baseline to 12-month follow-up, indicating that 5% of the MILD sample experienced a clinically relevant increase in pain-related disability. Six participants improved 11 or more points in the PDI and 31 participants reported no disability at all after twelve months.

Bivariate correlations between study variables at baseline and pain-related disability at the 12-month follow-up are reported in Table 3.

**Table 3:** Bi-variate associations between pain-related disability (PDI) at 12 months and independent variables at baseline

	**PDI at 12 months follow-up**	
**Independent variable at baseline**	**Pearson’s product–moment correlation r**	**p-value**
PDI n = 74	.667	<.001
Pain intensity n = 74	.453	<.001
SES n = 73	-.40	<.001
TSK n = 73	.299	.01
CAT n = 74	.209	.074
IES n = 74	.201	.086

*Note:* N is based on those participants with completed questionnaires from baseline and 12 months follow up.

PDI = Pain Disability Index (0–70), low scores indicate low disability.

Pain intensity (3 items 0–10), low scores indicate low pain intensity, control (1 item 0–10), low scores indicate low control.

SES = Self-Efficacy Scale (0–200), low scores indicate low efficacy expectations.

TSK = The Tampa Scale of Kinesiphobia (17–68), low scores indicate low fear.

CAT = The Coping Strategies Questionnaire (0–36). High scores indicate higher frequency of catastrophic thinking.

IES = Impact of Event Scale (0–75). High scores indicate severe symptoms.

The final multiple linear regression model with backward selection showed that pain-related disability at baseline was the only salient predictor of pain-related disability at the 12-month follow-up, Adjusted R^2^ .66, F(1, 69) = 139.8, p < .0001. Statistics for the initial and final models respectively are reported in Table 4.

**Table 4:** Results from the multiple linear regression (backward) analysis

		**PDI at 12 months follow-up**				
**Model**	**Predictors at baseline n = 73**	**B**	**β**	**95% CI for B**	**p-value**	**R**^**2**^
1	PDI	0.85	-0.87	0.67 to 1.04	<.001	.69
	Pain Intensity	-0.28	-0.05	-1.28 to 0.73	.58	
	SES	-0.03	-0.05	-0.10 to 0.05	.50	
	TSK	-0.10	-0.06	-0.34 to 0.147	.43	
	CAT	0.12	0.08	-0.11 to 0.35	.30	
	IES	-0.12	-0.12	-0.28 to 0.04	.15	
6	PDI	0.80	0.82	0.66 to 0.93	<.001	.67

Note:

PDI = Pain Disability Index (0–70), low scores indicate low disability.

Pain intensity (3 items 0–10), low scores indicate low pain intensity, control (1 item 0–10), low scores indicate low control.

SES = Self-Efficacy Scale (0–200), low scores indicate low efficacy expectations.

TSK = The Tampa Scale of Kinesiphobia (17–68), low scores indicate low fear.

CAT = The Coping Strategies Questionnaire (0–36). High scores indicate higher frequency of catastrophic thinking.

IES = Impact of Event Scale (0–75). High scores indicate severe symptoms.

## Discussion

This is one of the first studies intending to externally validate findings from previous explorative studies on prognostic factors in acute WAD in an initially self-selected MILD sample. The MILD sample reported consistently less imposition in all clinical variables compared to the MOD/SEV sample. The MILD sample decreased statistically in pain-related disability, pain catastrophising, and post traumatic stress symptoms over the first year after the accident, whereas functional self-efficacy and fear of movement/(re)injury increased. Pain intensity was low and stable. The reported changes were small on a group level and the clinical importance can be questioned. We found that 5% of the sample reported a clinically relevant deterioration in pain-related disability. Although very few they may represent those patients declining treatment in the acute phase, but reported to show up in health care later on.

Pain-related disability at baseline emerged as the only indicator of prognosis after 12 months. Hence the prediction model was not valid in the MILD sample except for the contribution of pain-related disability. Based on a systematic review and meta-analysis, Walton et al. [5] established a cut-off point of 5.5 of 10 on a visual analogue pain scale, with pain greater than this approaching a sixfold increase in the risk of persistent pain or disability over time. In our sample only 8 participants (11%) reported pain intensity ≥ 5 on the NRS at baseline, which could be one possible explanation of why pain did not emerge as a predictor in this sample.

An ongoing discussion is whether psychological variables measured in close connection to the accident are crucial for the prognosis, irrespective of levels of pain intensity and disability. It is proposed that the most important changes come off during the course of the first three months [4], and it is well established that psycho-social factors play an important role for the transition from subacute to chronic pain in other pain populations [28]. Our study points to that those classified with WAD grade I or II and subjectively mildly affected, already in the acute phase reported lower levels in psychological variables compared to those reported being moderately to highly affected and in need of treatment. The mechanisms behind the co-existence of low pain intensity, low pain-related disability and low imposition of psychological variables is of great interest but there was no possibility to rule out the temporal relationship between these variables in the acute stage in either of the samples. It has been proposed that psychological variables mediate the relationship between pain intensity and disability in WAD [7,8] and this mediation may be stronger when pain and disability are more severe. Theoretically, behavioral learning principles [29] may explain how experiences from previous accidents and pain conditions shape the current experience of the WAD and the development over time. For instance, recovery beliefs in the acute phase are associated with prognosis [3], and may be a result of such a previous learning process. Holm and colleagues [30] found that persons who stated they were less likely to make a full recovery were more likely to have high disability 6 months after the accident compared to persons who stated that they were likely to make a full recovery. We did not include any measure of recovery beliefs in the present study, but consider the possibility of positive recovery beliefs being a latent variable for the perception of being in no need of further treatments. The addition of this variable could have provided valuable information to our findings. The temporal dimensions and complex interaction between pain intensity, psychological variables and disability in the acute stage should be further elucidated in future studies. Another option for future research is to study the moderating effect of background characteristics on outcome. A somewhat surprising result was that the MILD reported proportionately lower levels of physical activity before the accident. A suggestion for future research is to study whether personal activity goals are related to perceived needs of treatment and pain-related disability. A previous study identified several activity related stressors in individuals with acute WAD [31] and knowledge is needed on how personal activity demands affect outcome and adaptation to the condition.

There are some important limitations with this study which are necessary to consider when interpreting the results. The sample was partly self-selected based on subjective statements of being mildly affected and in no need of treatment, which threatens the external validity of the results to those mildly affected without being under considerations for treatments within a randomized trial. Nevertheless, the systematically collected clinical data at baseline confirmed participants’ statements of being mildly affected, at least on a group level. Our prediction model was based on one point in time measures, whereas Sterling and colleagues [3] accentuate the value of inclusion of time-changing variables for the study of prognostic factors. We considered the inclusion of change scores in our predictive model, but did not find it motivated since changes over time were small. Instead we stayed with our initial research question of validation of previously identified predictors of prognosis in this particular subsample. The risk of floor effects in the pain intensity and pain-related disability measure should also be considered. Particularly in connection to clinically relevant improvements, which has been reported to 11 points or more on the PDI [27]. Thirty-one participants reported a score of 0 on the PDI at the 12-month follow-up. At first glance these were considered as recovered, but there is a possibility that the PDI may not be sensitive enough to capture variations in mild residual disability. Whether such variation is of clinical importance is though hard to rule out. The reliability of the pain intensity measure can also be questioned. Recalling average pain over a two-week window may introduce recall bias [32]. For future studies it would be more feasible to use a composite score of daily ratings on pain intensity [33]. The study of how the MOD/SEV sample developed over time and the validation of the prediction model in this sample would have been of great interest. However, only a small subsample (n = 19) was left with minimal treatments (standard self-management instructions), which did not allow for any multivariate linear regression analysis. Complete data from the RCT will be reported in the near future. Finally, it is worth noting that this study does not render any data on the causal influence on pain-related disability. Controlling for confounders is therefore not relevant at this stage [34].

## Conclusions

According to this study one can expect a good prognosis over the first year for the majority of individuals with WAD grade I or II who perceive themselves in no need of treatment in the acute stage. The MILD sample differed in outcomes i.e. self-reported pain intensity and pain-related disability from those who perceived themselves in need of treatment. They also reported lower imposition in psychological variables, and was characterised by higher proportions of WAD grade I, self-perceived good health status before the accident, and lower levels of physical activity and previous experience of a road accident. The prediction model was not valid for the MILD sample. Pain-related disability emerged as the only significant predictor. This study does not provide any established clinical prediction rule but points to that an early observation of individuals with elevated levels of pain-related disability is warranted, although they not perceive themselves in need of treatment.

## Abbreviations

MILD: Mildly affected sample; MOD/SEV: Moderately to severely affected sample.

## Competing interests

The authors declare that they have no competing interests.

## Authors’ contributions

PÅ participated in the design of the study, the statistical analyses of the study, and drafted the first version of the manuscript. AB participated in the design of the study, was responsible for the data collection, performed the statistical analysis, and contributed to the draft of the manuscript. AS participated in the design of the study, the statistical analyses of the study, and critically revised the manuscript. All authors read and approved the final manuscript.

## Pre-publication history

The pre-publication history for this paper can be accessed here:

http://www.biomedcentral.com/1471-2474/14/361/prepub

## References

[B1] Carroll L Holm L Hogg-Johnson S Course and prognostic factors for neck pain in whiplash-associated disorders (WAD): results of the Bone and Joint decade 2000–2010 Task Force on Neck Pain and Its associated Disorders Spine 2008 14 583 592 10.1097/BRS.0b013e3181643eb8 18204405

[B2] Hartling L Pickett W Brison R al e Derivation of a clinical decision rule for whiplash associated disorders aming individuals involved in rear-end collisions Accid Annal Prev 2002 14 531 539 10.1016/S0001-4575(01)00051-3 12067116

[B3] Sterling M Carroll L Kasch H Kamper S Stemper B Prognosis after whiplash injury Spine 2011 14 25S S330 S334 22020603 10.1097/BRS.0b013e3182388523

[B4] Kamper S Rebbeck T Maher C al e Course and prognostic factors factors of whiplash: a systematic review and meta-analysis Pain 2008 14 617 629 10.1016/j.pain.2008.02.019 18407412

[B5] Walton D Pretty J MacDermid J al e Risk factors for persistent problems following whiplash injury: results of a systematic review and meta-analysis J Orthop Sports Phys Ther 2009 14 334 350 10.2519/jospt.2009.2765 19411766

[B6] Williamson E Williams M Gates S Lamb SE A systematic literature review of psychological factors and the development of late whiplash syndrome Pain 2008 14 1–2 20 30 17570588 10.1016/j.pain.2007.04.035

[B7] Söderlund A Åsenlöf P The mediating role of self-efficacy expectations and fear of movement and (re)injury beliefs in two samples of acute pain Disabil Rehabil 2010 14 25 2118 2126 10.3109/09638288.2010.483036 20443673

[B8] Kamper SJ Maher CG Menezes Costa Lda C McAuley JH Hush JM Sterling M Does fear of movement mediate the relationship between pain intensity and disability in patients following whiplash injury? A prospective longitudinal study Pain 2012 14 113 119 10.1016/j.pain.2011.09.023 22054600

[B9] Sterling M Hendrikz J Kenardy J Compensation claim lodgement and health outcome developmental trajectories following whiplash injury: A prospective study Pain 2010 14 1 22 28 10.1016/j.pain.2010.02.013 20307934

[B10] Pate RR O'Neill JR Lobelo F The evolving definition of "sedentary" Exerc Sport Sci Rev 2008 14 4 173 178 10.1097/JES.0b013e3181877d1a 18815485

[B11] Spitzer W Skovron M Salmi L Cassidy D Duranceau J Suissa S Zeiss ES Scientific monograph of the Quebec Task Force on Whiplash-Associated Disorders: Redefining "Whiplash" and its management Spine 1995 14 7 73S 10.1097/00007632-199504151-00005 7604354

[B12] Tabachnick BG Fidell LS Using multivariate statistics 2001 4 Needham Heights, United State of America: Allyn & Bacon

[B13] Söderlund A Bring A Åsenlöf P A three-group study, internet-based, face-to-face based and standard- management after acute whiplash associated disorders (WAD) - choosing the most efficient and cost-effective treatment: study protocol of a randomized controlled trial BMC Musculoskelet Disord 2009 14 1 90 10.1186/1471-2474-10-90 19624833 PMC2722568

[B14] Pollard CA Preliminary validity study of Pain Disability Index Percept Mot Skills 1984 14 974 10.2466/pms.1984.59.3.974 6240632

[B15] Tait C Pollard CA Margolis RB Duckro PN The Pain Disability Index: Psychometric and Validity Data Arch Phys Med Rehabil 1987 14 438 441 3606368

[B16] Denison E Åsenlöf P Lindberg P Self-efficacy, fear avoidance, an pain intensity as predictors of disability in subacute and chronic musculoskeletal pain patients in primary health care Pain 2004 14 245 252 10.1016/j.pain.2004.07.001 15363867

[B17] Åsenlöf P Denison E Lindberg P Behavioural Goal Assessment in patients with persistent musculoskeletal pain Physiotherapy Theory and Practice 2004 14 243 254 10.1080/09593980490887957

[B18] The Swedish Centre on Health Technology Assessment (SBU) SBU Rehabilitation in chronic pain. A systematic review SBU report no 177/1+2 2010 Stockholm: SBU

[B19] Bergström G Bodin L Jensen I Linton S Nygren Å Long-term, non-specific spinal pain: reliable and valid subgroups of patients Beh Res Ther 2001 14 75 87 10.1016/S0005-7967(99)00175-8 11125725

[B20] Altmaier E Russell D Kao C Lehmann T Weinstein J Role of self-efficacy in rehabilitation outcome among chronic low back pain patients J Couns Psychol 1993 14 335 339

[B21] Vlaeyen J Kole-Snijders A Boeren R van Eek H Fear of movement/(re)injury in chronic low back pain in general practise:prognostic indicators of outcome B J Gen Pract 1995 14 519 523

[B22] Bunketorp L Carlsson J Kowalski J Stener-Victorin EJRM Jensen IL Evaluating the reliability of multi-item scales: a non-parametricapproach to the ordered categorical structure of data collected with the Swedish version of the Tampa Scale for Kinesiophopia and the Self-Efficacy Scale J Rehabil Med 2005 14 330 334 10.1080/16501970510036411 16208869

[B23] Rosenstiel AK Keefe FJ The use of coping strategies in low back pain patients: Relationship to patient characteristics and current adjustment Pain 1983 14 33 40 10.1016/0304-3959(83)90125-2 6226916

[B24] Jensen I Linton S Coping Strategies Questionnaire (CSQ): Reliability of the Swedish version of the CSQ Scan J Beh Ther 1993 14 139 145 10.1080/16506079309455940

[B25] Horowitz M Wilner N Alvarez W Impact of Event Scale: a measure of subjective stress Psychosom Med 1979 14 209 218 472086 10.1097/00006842-197905000-00004

[B26] Joseph S Psychometric evaluation of Horowitz's Impact of Event Scale: a review J Trauma Stress 2000 14 1 101 113 10.1023/A:1007777032063 10761177

[B27] Asenlof P Soderlund A A further investigation of the impolrtance of pain cognitions and behaivour in pain rehabilitation: longitudinal data suggest disability and fear of movement are most important Clin Rehabil 2010 14 5 422 230 10.1177/0269215509353264 20442254

[B28] Nicholas M Linton S Watson P Main C Early identification and management of psychological risk factors ("Yellow flags") in patients with low back pain: A reappraisal Phys Ther 2011 14 5 737 753 10.2522/ptj.20100224 21451099

[B29] Glanz K Rimer B Lewis F Health behavior and helath education 2002 3 San Francisco: John Wiley & Sons, Inc

[B30] Holm L Carroll L Cassidy J Skillgate E Ahlbom A Expectations for recovery important in the prognosis of whiplash injuries PLos Med 2008 14 5 760 767 10.1371/journal.pmed.0050105 PMC2375948 18479182

[B31] Bring A Soderlund A Wasteson E Asenlöf P Daily stressors in patients with acute whiplash associated disorders Disabil Rehabil 2012 14 1783 1789 10.3109/09638288.2012.662571 22512410

[B32] Gendreau M Hufford MR Stone AA Measuring clinical pain in chronic widespread pain: selected methodological issues Best Pract Res Clin Rheum 2003 14 4 575 592 10.1016/S1521-6942(03)00031-7 12849713

[B33] Jensen MP Lindsey RT Turner JA Romano JM The use of multiple-item scales for pain intensity measurement in chronic pain patients Pain 1996 14 34 40 10.1016/0304-3959(96)03078-3 8895229

[B34] Kamper SJ Hancock MJ Maher CG Optimal Designs for Prediction Studies of Whiplash Spine 2011 14 S268 S274 22020594 10.1097/BRS.0b013e3182388202

